# Thymic epithelial tumor treatment in Japan: analysis of hospital cancer registry and insurance claims data, 2012–2014

**DOI:** 10.1093/jjco/hyz167

**Published:** 2019-12-12

**Authors:** Hiroaki Kanemura, Tomohide Tamura, Naoki Nishimura, Daiki Kobayashi, Takahiro Higashi

**Affiliations:** 1 Department of Pulmonary Medicine, Thoracic Center, St. Luke’s International Hospital, Tokyo, Japan; 2 Department of Epidemiology, St. Luke’s International University Graduate School of Public Health, Tokyo, Japan; 3 Division of Health Services Research, Center for Cancer Control and Information Services, National Cancer Center, Tokyo, Japan

**Keywords:** chemotherapy, combined modality therapy, thymic carcinoma, thymoma

## Abstract

**Introduction:**

Thymic epithelial tumors are a rare type of neoplasm. Accordingly, it is difficult to perform phase III trials in patients with thymic epithelial tumors, and thus, no standard treatment has been established for these tumors. In this study, we aimed to investigate the current status of thymic epithelial tumor treatment in Japan.

**Methods:**

This retrospective observational study enrolled patients with thymic epithelial tumor whose data were recorded in a nationwide Hospital-based Cancer Registry that was linked with health insurance claims data for the registered patients between 2012 and 2014. The patients’ treatment details were obtained from a health insurance claims database.

**Results:**

A total of 813 patients with thymoma and 547 with thymic carcinoma were included in the analysis. Overall, 549 (67.5%) thymoma patients underwent surgical resection alone. Among patients with thymic carcinoma, 230 (42.0%) underwent initial surgery, and 124 (53.9%) received subsequent radiotherapy and chemotherapy. Chemotherapy regimens varied across the hospitals; overall, 21 and 22 regimens were used to treat thymoma and thymic carcinoma, respectively. Platinum-based combination regimens were predominantly selected for both diseases.

**Conclusions:**

This study revealed the real-world patterns of thymic epithelial tumor treatment in Japan. Although the nature of this study did not enable the determination of optimal treatment strategies, the simultaneous analysis of nationwide registry, insurance, efficacy and prognostic data may contribute to the establishment of a standard treatment strategy for rarely occurring cancers such as thymic epithelial tumor.

## Teaser

This study revealed the real-world patterns of thymic epithelial tumor treatment in Japan based on data from an Hospital-based Cancer Registry and a health insurance claims database.

## Introduction

Thymic epithelial tumors (TETs) are rarely occurring malignancies for which no standard treatment strategy (e.g. surgical resection, radiation therapy and chemotherapy and combinations thereof) has been established. Although the efficacy of primary surgical resection has been well-established for early-stage disease (1,2), no treatment has been established for advanced disease (3,4).

Currently, the treatment for a TET is determined by each physician based on expert opinions and the results of small clinical trials (1,5–13). The rarity of these tumors has made it difficult to conduct phase III trials in such settings (2,14). The International Thymic Malignancy Interest Group has created a global database to record data from Japan (3,15), USA, Italy, Germany (4) and China (16,17). However, this database predominantly includes cases treated with surgical resection but not cases of unresectable advanced disease, especially those involving distant metastasis of thymic carcinoma. Additionally, the guidelines for TET published by the National Comprehensive Cancer Network (NCCN) and European Society for Medical Oncology (ESMO) differ in several aspects (18,19). For example, the NCCN guideline describes 15 chemotherapy regimens, including second-line regimens, whereas the ESMO guideline describes only seven chemotherapy regimens and no second-line regimens. Therefore, this study aimed to investigate the current TET treatment situation in Japan.

## Materials and methods

### Data source

This retrospective observational study was based on a nationwide Japanese database. The main purpose of the Hospital-based Cancer Registry (HBCR) is to evaluate and improve the quality of care for patients with cancer (20). As of 2015, the Center for Cancer Control and Information Services of the National Cancer Center collected and managed HBCR data from 427 cancer care hospitals designated by the Ministry of Health, Labor and Welfare (21). These data may cover more than 67% of all newly diagnosed cancer patients (20).

The HBCR records information on newly encountered patients with malignant, intracranial borderline and benign tumors at the registering hospital. The recorded information includes (i) demographic characteristics, including the sex and date of birth, tumor site and histology codes according to the International Classification of Diseases for Oncology, third edition (ICD-O-3) (22); (ii) clinical and pathologic Tumor, node, metastasis (TNM) stage according to the Union for International Cancer Control (23); (iii) diagnosis date; and (iv) treatment details (20). After the registration, the National Cancer Center calls for the followed up data with health insurance claims data up to the end of the next year of diagnosis, which includes a maximum of 2 years from the date of diagnosis to monitor the quality and patterns of care nationwide. The health insurance claims database includes details regarding the care provided to each patient, including the health services and prescriptions provided, together with the corresponding dates (24). The National Cancer Center collects health insurance claims data from each hospital in a manner that is linkable with the HBCR.

We used the data for patients diagnosed from 2012 to 2014 who received treatment from hospitals that responded to the National Cancer Center call for data. We created a list of TET patients from the HBCR and analyzed the treatment patterns using information obtained from the health insurance claims database (20). These data were anonymized.

### Pathology and staging

The database recorded pathology data that were evaluated according to the ICD-O-3 classification (25). HBCR recorded tumor site and histology codes based on the ICD-O-3. The definition of ‘not otherwise specified (NOS)’ in ICD-O-3 is the category that cannot be classified into any definite code in ICD-O-3. Cases involving surgery were evaluated using surgical specimens, whereas biopsy specimens were used for other cases. This classification method was based on the Summary Stage created by the California Tumor Registry Office and the Surveillance, Epidemiology and End Results plan developed by the National Cancer Institute (26). Tumors were classified as Stage 0: *in situ*, I: localized, II: regional lymph nodes, III: regional by direct extension with or without regional lymph node metastasis and IV: distant metastasis.

### Patient selection

We extracted data on all patients diagnosed with thymoma or thymic carcinoma between 1 January 2012, and 31 December 2014 from the HBCR. The following codes were used for patient identification: (i) registered site on cancer, thymus (C37) and (ii) ICD-O-3 histology codes 8020, 8023, 8033, 8070, 8082, 8123, 8140, 8200, 8260, 8310, 8430, 8480, 8560, 8576, 8580–8585 and 8586 with a behavior code of 3 (i.e. malignant tumor). The data were limited to patients who were diagnosed and consequently treated at the same hospital. Patients who moved and initiated treatment at other hospitals were excluded.

### Hospital type

The hospital types were generally identified with respect to institutional names. We categorized a hospital as a cancer center or university hospital, if that hospital has ‘cancer center’ or a university’s name, in the name of the institution, respectively. The others were categorized as municipal hospitals.

### Compliance rate

It is defined as the proportion of regimens described in NCCN or ESMO guidelines to the total number of regimens.

### Ethical consideration

This study protocol and the use of the HBCR and health insurance claims database were approved by the Institutional Review Board and Data Use Committee at the National Cancer Center (Registration number: 2013-081; Approval date: 25 July 2013). The study was conducted in accordance with the principles of the Declaration of Helsinki (as revised in Fortaleza, Brazil in 2013). In publications analyzing the aforementioned data, the number of cases must be masked when <10, based on HBCR’s privacy policy. We categorized the group ‘<10’ as ‘1–3’, ‘4–6’ and ‘7–9’ without percentage.

### Statistical Analysis

Differences between the groups were assessed using the chi-square test for categorical variables and the Mann–Whitney U test and Kruskal–Wallis test for continuous variables. All tests for significance were two-tailed, with an α-value of 0.05. The Stata® version 15.0 software (StataCorp, College Station, TX, USA) was used for all statistical analyses.

## Results

### Patient characteristics

**Figure 1.**
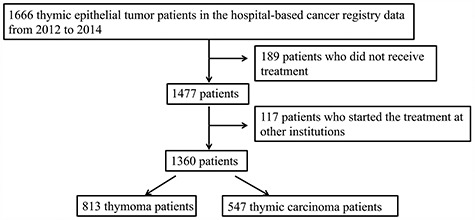
Flow chart of patient inclusion.

A total of 1666 TET patients were diagnosed with thymoma or thymic carcinoma. After excluding patients who received treatment from other hospitals, 813 thymoma patients and 547 thymic carcinoma patients were included in the analysis (Fig. 1). Of the thymoma patients, 389 (47.8%) were male, and the median age at diagnosis was 63 years. Of the 466 patients with a known disease stage, 295 (63.3%) had stage I disease, among whom 283 (95.9%) underwent surgery as the primary treatment. Of the thymic carcinoma patients, 362 (66.2%) were male, a significantly higher frequency than that observed among thymoma cases (*P* < 0.01). The median age at diagnosis was 65 years; again, this was significantly higher than that of thymoma patients (*P* < 0.01) (Table 1).

**Table 1:** Patient characteristics

Characteristic	All patients (*N* = 1360)	Thymoma (*N* = 813), *N* (%) or *N* (range)	Thymic carcinoma (*N* = 547), *N* (%) or *N* (range)	*P*-value
Age in years: median (range)		63 (16–90)	65 (20–85)	<0.01
Sex: number (%)	Male	389 (47.8)	362 (66.2)	<0.01
	Female	424 (52.2)	185 (33.8)	
Hospital type: number (%)	Municipal hospital	388 (47.7)	279 (51.0)	0.46
	Cancer center	87 (10.7)	58 (10.6)	
	University hospital	338 (41.6)	210 (38.4)	
Stage: number (%)	I	295 (36.3)	89 (16.3)	<0.01
	II	2 (0.2)	6 (1.1)	
	III	127 (15.6)	105 (19.2)	
	IV	42 (8.3)	118 (21.6)	
	No data	347 (42.6)	229 (41.9)	
WHO classification: number (%)	A	54 (6.6)		
	AB	162 (19.9)		
	B1	154 (18.9)		
	B2	174 (21.4)		
	B3	121 (14.9)		
	NOS	148 (18.2)		
Pathology of thymic cancer: number (%)	Squamous cell carcinoma		282 (51.6)	
	Adenocarcinoma		12 (2.2)	
	Others		20 (3.7)	
	Basaloid carcinoma		7–9	
	Mucoepidermoid carcinoma		7–9	
	Sarcomatoid carcinoma		1–3	
	Undifferentiated carcinoma		4–6	
	NOS		233 (42.6)	

WHO: World Health Organization, NOS: not otherwise specified.

Of the 318 patients with thymic carcinoma and a known disease stage, 223 (70.1%) had stage III or IV disease, indicating that this type of malignancy was significantly more frequently diagnosed at a later stage than thymoma (*P* < 0.01) (Table 1). Sixty-six (29.6%) patients with thymic carcinoma underwent surgery as the primary treatment, whereas 157 (70.4%) received radiotherapy or chemotherapy (Table 2).

**Table 2:** Patient characteristics by initial treatment

Characteristic	Thymoma (813)	Surgery (689), *N* (%) or *N* (range)	Radiotherapy (21), *N* (%) or *N* (range)	Chemotherapy (103), *N* (%) or *N* (range)	*P*-value
Age in years: median (range)		62 (16–87)	73 (43–90)	63 (23–81)	<0.01
Sex: number (%)	Male	317 (46.0)	8 (38.1)	64 (62.1)	<0.01
	Female	372 (54.0)	13 (61.9)	39 (37.9)	
Stage: number (%)	I	283 (41.1)	1–3	9 (8.7)	<0.01
	II	2 (0.3)	0	0	
	III	90 (13.1)	4–6	33 (32.0)	
	IV	15 (2.2)	4–6	21 (20.4)	
	No data	299 (43.4)	7–9	40 (38.8)	
WHO classification: number (%)	A	51 (7.4)	1–3	1–3	<0.01
	AB	155 (22.5)	0	7–9	
	B1	140 (20.3)	1–3	11 (10.7)	
	B2	150 (21.8)	4–6	19 (18.5)	
	B3	97 (14.1)	4–6	19 (18.5)	
	NOS	96 (13.9)	4–6	46 (44.7)	
	Thymic carcinoma (547)	Surgery (230)	Radiotherapy (67)	Chemotherapy (250)	
Age in years: median (range)		66 (20–85)	68 (36–85)	64 (22–84)	<0.01
Sex: number (%)	Male	141 (61.3)	46 (68.7)	175 (70.0)	0.12
	Female	89 (38.7)	21 (31.3)	75 (30.0)	
Stage: number (%)	I	70 (30.4)	7 (10.4)	12 (4.8)	<0.01
	II	3 (1.3)	1–3	2 (0.8)	
	III	47 (20.4)	7–9	50 (20.0)	
	IV	19 (8.3)	19 (28.4)	80 (32.0)	
	No data	91 (39.6)	32 (47.8)	106 (42.4)	

**Table 3:** Regimens in patients with chemotherapy

Thymoma (*N* = 135)			Frequency *N* (%)
Single agent			6 (4.4)
	AMR		1–3
	GEM^a^		1–3
	PEM^a^		1-3
	Sirolimus		1–3
	EPI		1–3
Platinum combination	CDDP combination		75 (55.6)
		DXR + CPA+VCR^b^	37 (27.4)
		DXR	13 (9.6)
		Others	25 (18.5)
		DXR+CPA^b^	7-9
		AMR	4-6
		ETP^a^	4-6
		DXR + ETP+VCR^c^	1-3
		5-FU	1–3
		S-1	1–3
		VNR	1–3
		CPT-11	1–3
	CBDCA combination		54 (40.0)
		PTX^b^	47 (34.8)
		Others	7 (5.2)
		DOC	1–3
		ETP	1–3
		DXR	1–3
		GEM	1–3
		S-1	1–3
Compliance rate (%)			74.1
Thymic carcinoma (*N* = 316)			Frequency *N* (%)
Single agent			19 (6.0)
	S-1		7–9
	DOC		4–6
	AMR		1–3
	PTX^a^		1-3
	THP		1–3
Platinum combination	CDDP combination		81 (25.6)
		DXR + CPA + VCR^b^	39 (12.3)
		ETP^b^	13 (4.1)
		Others	29 (9.2)
		DOC	7–9
		CPT-11	4–6
		DXR + CPA^b^	4-6
		VNR	4-6
		S-1	1–3
		DXR + ETP + VCR^c^	1-3
		DXR	1-3
		5-FU	1–3
	CBDCA combination		215 (68.0)
		PTX^b^	195 (61.7)
		Others	20 (6.3)
		GEM	7–9
		S-1	4–6
		DXR	4–6
		ETP	1–3
		DOC	1–3
Others	DOC + nedaplatin		1–3
Compliance rate (%)			80.7

AMR: amurubicin, GEM: gemcitabine, PEM: pemetrexed, EPI: epirubicin, CDDP: cisplatin, DXR: doxorubicin, CPA: cyclophosphamide, VCR: vincristine, ETP: etoposide, 5-FU: fluorouracil, S-1: tegafur/gimeracil/oteracil, VNR: vinorelbine, CBDCA: carboplatin, PTX: paclitaxel, DOC: docetaxel, THP: pirarubicin, NCCN: National Comprehensive Cancer Network, ESMO: European Society for Medical Oncology, CPT-11: irrinotecan.

^a^Regimens described only in NCCN guidelines.

^b^Regimens described in NCCN and ESMO guidelines.

^c^Regimens described only in ESMO guidelines.

**Table 4:** Regimens in patients with chemotherapy by hospital type

Thymoma (*N* = 135)	Municipal hospital (*N* = 68), *N* (%)	Cancer center (*N* = 18), *N* (%)	University hospital (*N* = 49), *N* (%)
Monotherapy	4 (5.9)	2 (11.1)	0
AMR	1 (1.5)	1 (5.6)	
GEM^a^		1 (5.6)	
PEM^a^	1 (1.5)		
Sirolimus	1 (1.5)		
EPI	1 (1.5)		
CBDCA + PTX^b^	20 (29.4)	7–9	20 (40.8)
CDDP + DXR + CPA + VCR^b^	17 (25.0)	1–3	18 (36.7)
Others	27 (39.7)	7 (38.9)	11 (22.4)
CDDP + DXR	10 (14.7)	1–3	1–3
CDDP + DXR + CPA^b^	4–6		1–3
CDDP + AMR		4–6	1–3
CDDP + ETP^a^	1–3	1–3	1–3
CDDP + DXR + ETP + VCR^c^	1–3		1–3
CDDP + 5-FU	1–3		
CBDCA + DOC	1–3		1–3
CBDCA + ETP	1–3		
CDDP + S-1	1–3		
CDDP + VNR	1–3		
CDDP + CPT-11	1–3		
CBDCA + DXR			1–3
CBDCA + GEM	1–3		
CBDCA + S-1	1–3		
Compliance rate (%)	66.2	61.1	87.8
Thymic carcinoma (*N* = 316)	Municipal hospital (*N* = 156)	Cancer center (*N* = 30)	University hospital (*N* = 130)
Monotherapy	10 (6.4)	0	9 (6.9)
S-1	4–6		4–6
DOC	1–3		1–3
AMR			1–3
PTX^a^	1–3		
THP	1–3		
CBDCA + PTX^b^	88 (56.4)	23 (76.7)	84 (64.6)
CDDP + DXR + CPA + VCR^b^	22 (14.1)	1 (3.3)	16 (12.3)
Others	36 (23.1)	6 (20.0)	21 (16.2)
CDDP + ETP^b^	4–6	4–6	1–3
CDDP + DOC	4–6		
CBDCA + GEM	4–6		1–3
CDDP + CPT-11	4–6		1–3
CBDCA + S-1	1–3		4–6
CDDP + DXR + CPA^b^	4-6		
CDDP + VNR	1–3		1–3
CBDCA + DXR	1–3		1–3
CDDP + S-1	1–3		1–3
CBDCA + ETP			1–3
CDDP + DXR + ETP + VCR^c^	1–3	1–3	
CDDP + DXR	1–3		4–6
CDDP + 5-FU	1–3		
CBDCA + DOC			1–3
DOC + nedaplatin	1–3		
Compliance rate (%)	78.8	100.0	78.4

^a^Regimens described only in NCCN guidelines.

^b^Regimens described in NCCN and ESMO guidelines.

^c^Regimens described only in ESMO guidelines.

Table 2 shows the patients’ backgrounds according to the initial treatment type. In both disease groups, patients receiving initial radiotherapy were significantly older than those receiving other treatments (*P* < 0.01). Most patients with stage I disease underwent initial surgery, whereas those with advanced-stage (III or IV) disease tended to receive initial radiotherapy or chemotherapy. Regarding thymoma pathology, patients with World Health Organization (WHO) classification A, AB and B1 disease underwent initial surgery, whereas those with higher cellular atypia and invasiveness (B2 and B3) tended to receive initial radiotherapy or chemotherapy.

A significant difference in the rate of surgery was observed among thymoma patients with respect to hospital type but not among thymic carcinoma patients (Table S1). The rate of surgery was the lowest in cancer centers. In the operative method (open surgery or thoracoscopic surgery), no difference existed between both thymoma and thymic carcinoma patients (Table S2).

### Treatment pattern

#### Thymoma

**Figure 2.**
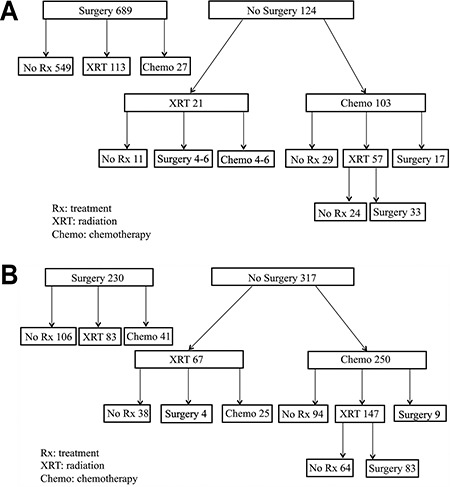
(a) Treatment patterns in patients with thymoma. (b) Treatment patterns in patients with thymic carcinoma. Rx: treatment, XRT: radiation, Chemo: chemotherapy.

Of the 689 patients who underwent initial surgery, 549 (79.7%) did not receive subsequent treatment (Fig. 2). Furthermore, 124 patients did not undergo surgical resection, 103 (83.0%) patients received initial chemotherapy and 21 (16.9%) received initial radiotherapy. Of the patients who received initial chemotherapy, 29 (28.2%) did not receive further treatment, 17 (16.5%) underwent subsequent surgery and 57 (55.3%) received subsequent radiotherapy. Thirty-three (57.9%) of these patients subsequently underwent surgery. Seventeen patients underwent neoadjuvant chemotherapy. The regimens have been listed in Table S3. Additionally, five patients underwent radiotherapy before surgery.

#### Thymic carcinoma

Of the 230 patients who underwent surgery, 106 (46.1%) did not receive subsequent treatment; this proportion was lower than that observed for thymoma patients (Fig. 2). Three hundred and seventeen patients did not undergo surgical resection. A total of 250 patients (78.9%) received initial chemotherapy, and 67 (21.1%) received initial radiotherapy. Of those who received initial chemotherapy, 94 (37.6%) did not receive further treatment, and 147 (58.8%) received subsequent radiotherapy. Eighty-three (56.5%) of the latter patients subsequently underwent surgery. Nine patients underwent neoadjuvant chemotherapy. The regimens have been listed in Table S3. In addition, four patients underwent radiotherapy before surgery.

### Chemotherapy regimens

#### Thymoma

A total of 135 thymoma patients received 21 chemotherapy regimens. Nearly all cases (129; 95.6%) received platinum combination regimens. The most commonly used regimens were carboplatin + paclitaxel (CBDCA + PTX), cisplatin + doxorubicin  + cyclophosphamide + vincristine (CDDP + DXR + CPA + VCR) and cisplatin + doxorubicin (CDDP + DXR). Amurubicin (AMR), gemcitabine (GEM), pemetrexed (PEM), sirolimus and epirubicin (EPI) monotherapies were used in a few cases. Overall, 74.1% of these chemotherapy regimens were described in the NCCN or ESMO guidelines (Table 3). According to hospital type, municipal hospitals used the highest number of regimens, whereas university hospitals did not use monotherapies. CBDCA + PTX was the most commonly used regimen across all hospital types. Cisplatin + amurubicin (CDDP + AMR) was used more frequently than CDDP + DXR + CPA + VCR only in cancer centers. Cancer centers had a compliance rate of 61.1%, which was the lowest across all hospital types (Table 4).

#### Thymic carcinoma

A total of 316 thymic carcinoma patients underwent 22 chemotherapy regimens. Most cases (296; 93.7%) were treated using platinum combination regimens. CBDCA + PTX (195; 61.7%) was the most commonly used regimen. Monotherapies, including tegafur/gimeracil/oteracil (S-1), docetaxel, AMR, PTX and pirarubicin (THP), were used in 19 (6.0%) cases. Overall, 80.7% of these regimens complied with the NCCN or ESMO guidelines (Table 3). According to hospital type, municipal hospitals used the greatest number of regimens, whereas cancer centers did not use monotherapies. Across all hospital types, CBDCA + PTX was the most commonly used regimen. CDDP + DXR + CPA + VCR was used in municipal and university hospitals, but rarely in cancer centers. Cisplatin + etoposide (CDDP + ETP) was more commonly used in cancer centers than in other hospital types. The compliance rate for cancer centers was 100.0%, which was the highest across all hospital types (Table 4).

## Discussion

This study is the first to describe the real-world treatment patterns for TETs, based on data from an HBCR and a health insurance claims database. This analysis indicated that thymoma tended to be diagnosed at earlier stages and that the majority of patients (549; 67.5%) underwent primary surgery. CDDP + DXR + CPA + VCR was the most commonly used chemotherapy regimen for thymoma. According to a report from an Italian research group in 1990, this regimen, which has been used for more than two decades, has an overall response rate of 91.8% and a complete response rate of 43% (27). Notably, CBDCA + PTX was also frequently used for thymoma. However, the efficacy data associated with this regimen are limited when compared with the data for thymic carcinoma, with a reported overall response rate of 42.9% (28).

Thymic carcinoma tended to be diagnosed more frequently in advanced stages, and 106 (19.4%) affected patients underwent primary surgery, whereas 83 (15.2%) received initial chemotherapy and subsequent radiotherapy and surgery. The most commonly used regimen was CBDCA + PTX; however, these data were based on phase II trials conducted in 2011 and 2015, which reported an overall response rate of 21.7% (28,29). CDDP + DXR + CPA + VCR was also used for thymic carcinoma; however, data on the use of this regimen for thymic carcinoma are limited to retrospective analyses or small case series (30).

The uses of AMR, irinotecan and nedaplatin (31), which were all developed in Japan, are unique to this country. Currently, the efficacy of CDDP + AMR for thymoma is being evaluated in a clinical trial by the West Japan Oncology Group 5509 L (UMIN000003933). The use of AMR for thymic carcinoma is based on the findings of a retrospective observational study (32). Notably, 13 of the 21 regimens used for thymoma (18,19) and 16 of the 22 regimens used for thymic carcinoma in this analysis were not described in the NCCN and ESMO guidelines.

An analysis by hospital type revealed several differences between cancer centers and other hospital types. Regimens comprising new drugs tended to be used more frequently in cancer centers. As shown in this study, most chemotherapy regimens used to treat TETs involved a combination of old and low-cost drugs. However, pharmaceutical companies have not conducted clinical trials intended to support the approval of old drugs for TETs. In Japan, drug approval can be obtained using a public knowledge-based application (33), which is based on the international use of a drug or the presence of sufficient medical literature to prove its safety and efficacy in the absence of a clinical trial. Although nationwide registry, insurance and efficacy data are all sources of domestic data, the associated analyses may provide useful information for public knowledge-based application. However, newer agents should be subjected to a prospective global clinical trial prior to approval. Global clinical trials for several new drugs, such as pembrolizumab, sunitinib and everolimus, are currently underway (34–37).

Our study had several limitations. First, we lacked data on patient performance status and presence of comorbidities and therefore could not identify the main factor(s) associated with treatment decision-making. Variations in treatment choice may partially be due to the patients’ conditions. Second, the data were obtained only from hospitals where patients were diagnosed and started their treatment. If patients were transferred after the start of therapy or concurrently treated in other hospitals, the health services provided in the other hospitals were not included in the data. Third, the maximum follow-up period was 2 years, a relatively short period given the overall survival of patients with thymic tumors. When treatment was provided over an extended period, the data analyzed for these patients may have been assessed inaccurately in the results. Fourth, this was a descriptive study of voluntarily participating hospitals. Although the size of the data was larger than that of other prior studies assessing nonspecialized hospitals, nonparticipating hospitals might have different practice patterns. Fifth, the ICD-O-3 system includes the codes for thymoma of borderline malignancy, but they are not reportable to HBCR. Since tumors of the thymus are reportable to HBCR only when they are deemed malignant, it is possible that some thymomas diagnosed as borderline malignancies were not included in the HBCR. Nevertheless, the WHO classification of thymoma in the previous edition suggests that thymic tumors are automatically designed as malignant (38); in addition, the current version codes all thymoma as malignant (25). Sixth, pathology classification and stage included unknown or NOS data in many parts, which were excluded from the statistical analyses.

In conclusion, we analyzed the real-world treatment patterns associated with TETs in Japan. For rarely occurring cancers, such as TETs, analyses of nationwide registry, insurance, efficacy and prognostic data may contribute to the establishment of a standard treatment strategy.

## Supplementary Material

Table_S1_hyz167

Table_S2_hyz167

Table_S3_hyz167
