# Supplementary material for: Thymic epithelial tumor treatment in Japan: analysis of hospital cancer registry and insurance claims data, 2012–2014
Source: Jpn J Clin Oncol. 2019 Dec 12;50(3):310–7. doi: 10.1093/jjco/hyz167 (PMC7061247; doi:10.1093/jjco/hyz167)
Supplement: Table_S1_hyz167 [file table_s1_hyz167.doc]

**Table S1. Comparison of patients with respect to surgery and hospital type**

| Thymoma (N = 813) | | | | |
| --- | --- | --- | --- | --- |
|  | Municipal hospital  (N = 388) N (%) | Cancer center  (N = 87) N (%) | University hospital  (N = 338) N (%) | p-value |
| Surgery | 245 (70.0) | 46 (62.2) | 232 (72.5) | 0.02 |
| No surgery | 143 (30.0) | 41 (37.8) | 106 (27.5) |  |
| Thymic carcinoma (N = 547) | | | | |
|  | Municipal hospital  (N = 279) N (%) | Cancer center  (N = 58) N (%) | University hospital  (N = 210) N (%) | p-value |
| Surgery | 167 (59.9) | 30 (51.7) | 129 (61.4) | 0.41 |
| No surgery | 112 (40.1) | 28 (48.3) | 81 (38.6) |  |
