# Supplementary material for: Thymic epithelial tumor treatment in Japan: analysis of hospital cancer registry and insurance claims data, 2012–2014
Source: Jpn J Clin Oncol. 2019 Dec 12;50(3):310–7. doi: 10.1093/jjco/hyz167 (PMC7061247; doi:10.1093/jjco/hyz167)
Supplement: Table_S2_hyz167 [file table_s2_hyz167.doc]

**Table S2. Comparison of operative methods in patients who underwent surgery with respect to hospital type**

| Thymoma (N = 774) | | | | |
| --- | --- | --- | --- | --- |
| Operative method | Municipal hospital  (N = 350) N (%) | Cancer center  (N = 74) N (%) | University hospital  (N = 320) N (%) | p-value |
| Open surgery | 245 (70.0) | 46 (62.2) | 232 (72.5) | 0.21 |
| Thoracoscopic surgery | 105 (30.0) | 28 (37.8) | 88 (27.5) |  |
| Thymic carcinoma (N = 326) | | | | |
| Operative method | Municipal hospital  (N = 167) N (%) | Cancer center  (N = 30) N (%) | University hospital  (N = 129) N (%) | p-value |
| Open surgery | 119 (71.3) | 23 (76.7) | 95 (73.6) | 0.80 |
| Thoracoscopic surgery | 48 (28.7) | 7 (23.3) | 34 (26.4) |  |
