# Supplementary material for: Thymic epithelial tumor treatment in Japan: analysis of hospital cancer registry and insurance claims data, 2012–2014
Source: Jpn J Clin Oncol. 2019 Dec 12;50(3):310–7. doi: 10.1093/jjco/hyz167 (PMC7061247; doi:10.1093/jjco/hyz167)
Supplement: Table_S3_hyz167 [file table_s3_hyz167.doc]

**Table S3. Regimens in patients with neoadjuvant chemotherapy**

| Thymoma (N = 17) |
| --- |
| CBDCA+PTX |
| CDDP+DXR+CPA+VCR |
| CDDP+DXR |
| CDDP+DXR+ETP+VCR |
| CDDP+DXR+CPA |
| CDDP+ETP |
| Thymic carcinoma (N = 9) |
| CDDP+DXR+CPA+VCR |
| CBDCA+PTX |
| CDDP+DXR |
| CDDP+DOC |
| S-1 |
| THP |
